# Overexpression of mutant HSP27 causes axonal neuropathy in mice

**DOI:** 10.1186/s12929-015-0154-y

**Published:** 2015-06-19

**Authors:** Jinho Lee, Sung-Chul Jung, Jaesoon Joo, Yu-Ri Choi, Hyo Won Moon, Geon Kwak, Ha Kyung Yeo, Ji-Su Lee, Hye-Jee Ahn, Namhee Jung, Sunhee Hwang, Jingeun Rheey, So-Youn Woo, Ji Yon Kim, Young Bin Hong, Byung-Ok Choi

**Affiliations:** Department of Neurology, Samsung Medical Center, Sungkyunkwan University School of Medicine, 81 Irwon-ro, Gangnam-gu, Seoul 135-710 Korea; Department of Biochemistry, Ewha Womans University School of Medicine, Seoul, Korea; Samsung Biomedical Research Institute, Samsung Advanced Institute of Technology, Seoul, Korea; Microbiology, Ewha Womans University School of Medicine, Seoul, Korea; Stem Cell & Regenerative Medicine Center, Samsung Medical Center, 81 Irwon-ro, Gangnam-gu, Seoul 135-710 Korea; Neuroscience center, Samsung Medical Center, Seoul, Korea

**Keywords:** Heat shock 27 kDa protein 1 (HSP27 or HSPB1), Charcot-Marie-Tooth disease (CMT), Distal hereditary motor neuropathy (dHMN), Axonopathy, Mouse model, Magnetic resonance image (MRI)

## Abstract

**Background:**

Mutations in heat shock 27 kDa protein 1 (HSP27 or HSPB1) cause distal hereditary motor neuropathy (dHMN) or Charcot-Marie-Tooth disease type 2 F (CMT2F) according to unknown factors. Mutant HSP27 proteins affect axonal transport by reducing acetylated tubulin.

**Results:**

We generated a transgenic mouse model overexpressing HSP27-S135F mutant protein driven by Cytomegalovirus (CMV) immediate early promoter. The mouse phenotype was similar to dHMN patients in that they exhibit motor neuropathy. To determine the phenotypic aberration of transgenic mice, behavior test, magnetic resonance imaging (MRI), electrophysiological study, and pathology were performed. Rotarod test showed that founder mice exhibited lowered motor performance. MRI also revealed marked fatty infiltration in the anterior and posterior compartments at calf level. Electrophysiologically, compound muscle action potential (CMAP) but not motor nerve conduction velocity (MNCV) was reduced in the transgenic mice. Toluidine staining with semi-thin section of sciatic nerve showed the ratio of large myelinated axon fiber was reduced, which might cause reduced locomotion in the transgenic mice. Electron microscopy also revealed abundant aberrant myelination. Immunohistochemically, neuronal dysfunctions included elevated level of phosphorylated neurofilament and reduced level of acetylated tubulin in the sural nerve of transgenic mice. There was no additional phenotype besides motor neuronal defects.

**Conclusions:**

Overexpression of HSP27-S135F protein causes peripheral neuropathy. The mouse model can be applied to future development of therapeutic strategies for dHMN or CMT2F.

**Electronic supplementary material:**

The online version of this article (doi:10.1186/s12929-015-0154-y) contains supplementary material, which is available to authorized users.

## Background

Mutations in heat shock 27 kDa protein 1 (HSP27, also called HSPB1) are associated with hereditary peripheral neuropathy [1–4]. The clinical aspects of the patients are heterogeneous and include motor neuropathy or sensory neuropathy with various onset ages, resulting in distal hereditary motor neuropathy (dHMN) type II (OMIM 608634) or Charcot-Marie-Tooth disease (CMT) type 2 F (OMIM 606595) [1–4].

HSP27 is located in chromosome 7q11-q21 and encodes a 27 kDa protein possessing an α-crystallin domain [5]. HSP27 protects cells from stresses and is associated with cell motility and cytoskeletal stabilization [6–10]. The expression of HSP27 ameliorates neurodegenerative diseases, such as Alzheimer’s disease [11, 12], Parkinson disease [13], and amyotrophic lateral sclerosis [14]. On the other hand, mutation in HSP27 deteriorates axonal transport in the peripheral nervous system [15]. Mutant HSP27 protein reduces neuronal cell viability and impairs neurofilament assembly [1, 16]. In addition, the expression of mutant HSP27 reduces acetylated α-tubulin in dorsal root ganglion cell (DRG) in mouse model, resulting in reduced mitochondrial movement and dysfunction of axon cytoskeleton and axonal transport [17].

So far, eight mutations in HSP27 are reported: L99M, R127W, S135F, R136W, R140G, T151I, P182S, and P182L. Intriguingly, the Ser135Phe mutation, which is the best characterized mutation, is associated with both CMT2F and dHMN [1]. This implies that various clinical phenotypes such as involvement of sensory neuron, onset age, and severity caused by the same mutation, might be dependent on the genetic background.

Currently, several mouse models (FVB/N strain) have been developed for HSP27 mutations including Pro182Leu [18], Ser135Phe [18], and Arg136Trp [19]. The phenotypes of these mice are similar to patients who develop late-onset motor neuropathy. Since the clinical symptoms of patients are heterogeneous according to genetic background, various models might be required for further study.

In this study, we generated a transgenic mouse (C57BL6/J strain) expressing S135F mutation in HSP27 to further investigate the clinical aspects and development of therapeutic strategies.

## Methods

### Preparation of HSP27 construct

To obtain human HSP27 gene, total mRNA from HEK 293 cells was used as the template for cDNA synthesis and PCR amplification. The amplified gene was cloned into the pcDNA3.1(+) vector, where CMV promoter drives the expression of the gene, for recombination and expression in mammalian cells. To generate the S135F mutant gene, site-directed mutagenesis was performed using the QuikChange Site-Directed Mutagenesis Kit (Stratagene, La Jolla, CA). All the sequences were confirmed by capillary sequencing.

### Generation of transgenic mouse model

To establish a mouse model for HSP27-S135F mutation, pcDNA3.1-HSP27-S135F was injected into fertilized eggs (C57BL6/J strain). The eggs were implanted into surrogate female mice. Seventy-two mice were generated and 12 harbored the HSP27-S135F construct. All experiments were conducted according to protocols approved by the Institutional Animal Care and Use Committees of Samsung Medical Center.

### Rotarod test

To evaluate motor coordination and balance of HSP27-S135F transgenic mice, the rotarod test was performed on a 3 cm horizontal rotating rod (2 m/min). To adapt to the test, the mice were pre-trained for one week. Testing was for a maximum of 7 min.

### Electrophysiological study

Ten control and transgenic mice, aged 7 months and weighing 25–30 g, were used for the electrophysiological study. The mice were anesthetized with 50 mg/kg Zoletil (Virbac, Seoul, Korea) intraperitoneally and the fur from the distal back and the hind limbs was completely removed. Nerve conduction study (NCS) was performed using Nicolet VikingQuest (Natus Medical, San Carlos, CA) [20]. The compound motor action potential (CMAP) amplitudes and motor nerve conduction velocity (MNCV) were determined.

### Magnetic resonance imaging (MRI)

*In vivo* monitoring of mouse hind limbs and muscle damage was performed using a Biospec 7.0 Tesla 30 cm horizontal bore scanner with Paravision 5.1 software (Bruker Biospin MRI GmbH, Germany) as detailed [21]. Briefly, Bruker four-element ^1^H volume coil array and Bruker 72 mm linear-volume coil were used as the receiver and the transmitter, respectively. Aaxial, mid-sagittal, and coronal scout rapid acquisition with fast low angle shot imaging was used to localize the leg, and high resolution T2-weighted images in the cross-sectional view were acquired with TR/TE (repetition time/echo time) = 5000/32 ms, field of view = 30 × 30 mm^2^, matrix size = 250 × 250, slice thickness = 0. 5 mm without a gap.

### Electron microscopy

The distal sural nerve was biopsied from mice at 10 months, and pathological examinations of affected individuals included light and electron microscopic analyses. Specimens were individually fixed in 2 % glutaraldehyde in 25 mM cacodylate buffer. Semithin sections were stained with toluidine blue and ultra-thin cut samples were contrasted with uranyl acetate and lead citrate.

### Immunohistochemistry and western blotting

Tissues from sciatic and median/ulnar nerve of the mice were analyzed for tubulin acetylation and neurofilament phosphorylation. Formalin-fixed sections were stained with hematoxylin and eosin (H&E). Anti-phospho-neurofilament and acetylated-α-tubulin antibody (Abcan, Cambridge, UK) were used. Standard Western blotting was performed using anti-tubulin (Abcam), anti-β-actin antibody, anti-mouse secondary antibody, and anti-rabbit secondary antibody (Sigma-Aldrich, St. Louis, MO) as well as ECL plus Western blotting substrate (Thermo Scientific, Rockford, IL). Band intensities were calculated by Image J program (www.imagej.nih.gov) then the ratio of the band intensity of acetylated-α-tubulin over α-tubulin and phospho-neurofilament over neurofilament was calculated.

### Statistical analysis

All animals were studied with a blind test. Comparison between normal and HSP27-S135F mice were made by Student’s *t*-test. P < 0.05 was considered statistically significant.

## Results

### HSP27-S135F transgenic mice exhibit reduced motor function

**Fig. 1:**
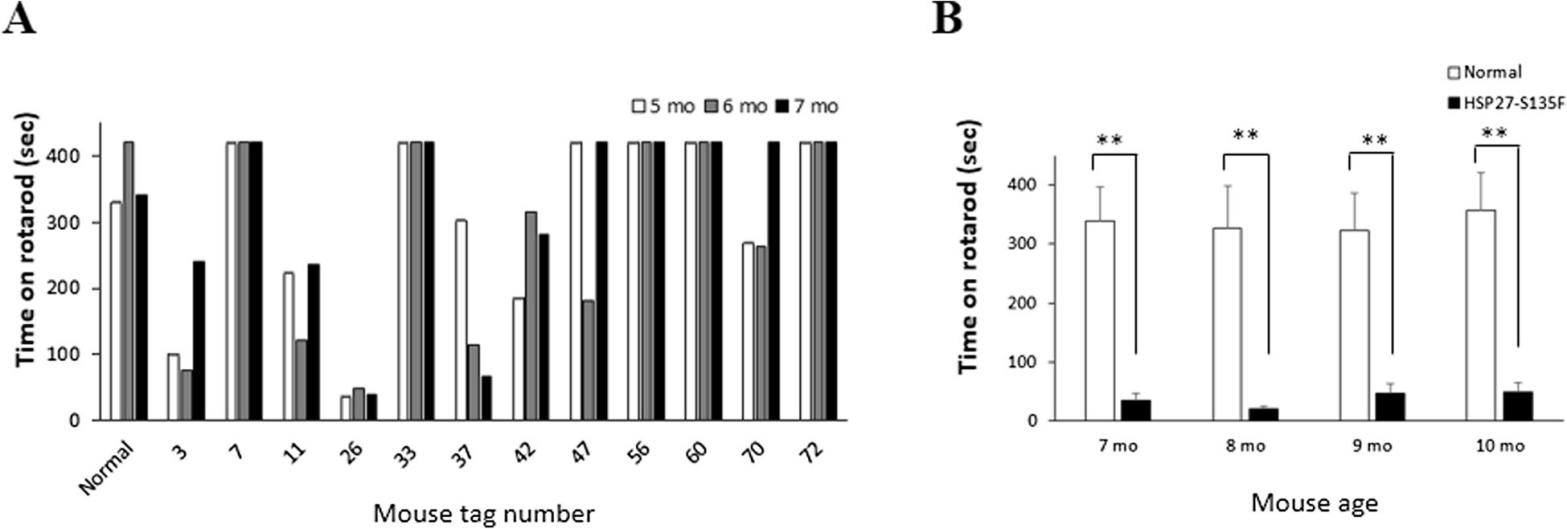
Reduced motor function in HSP27-S135F mouse. Rotarod test were applied to measure motor function of mice. Reduced performance was observed from both the founders from 5 months of age (**a**) and siblings of #11 and #26 from 7 months of age (**b**). **, *p* < 0.01

Wild type and S135F mutant HSP27 gene was cloned as described in Materials and Methods. Expression of the genes was confirmed in HEK293 and NSC34 cells (Additional file 1: Figure S1). To generate HSP27-S135F transgenic mice, pcDNA3.1-HSP27-S135F was injected into fertilized eggs. Twelve transgenic mice were confirmed to have the HSP27-S135F construct. From 5 months, motor function was assessed using the rotarod test. Compared with age matched control mice group (n = 10), several transgenic mice exhibited reduced motor performance (Fig. 1a). Upon completion of the tests, two mice (#11 and #26) were chosen as founders for further studies. Rotarod testing of the founders’ siblings also showed the same results (Fig. 1b). Measurement of grip strength on the progeny (F1) also demonstrated reduced motor function in the transgenic mice (Additional file 2: Figure S2). Besides the motor function, all the mice exhibited normal phenotype, except that #60 had white fur on chest, which was transmitted to its siblings.

### MRI demonstration of fatty infiltration in lower extremities

**Fig. 2:**
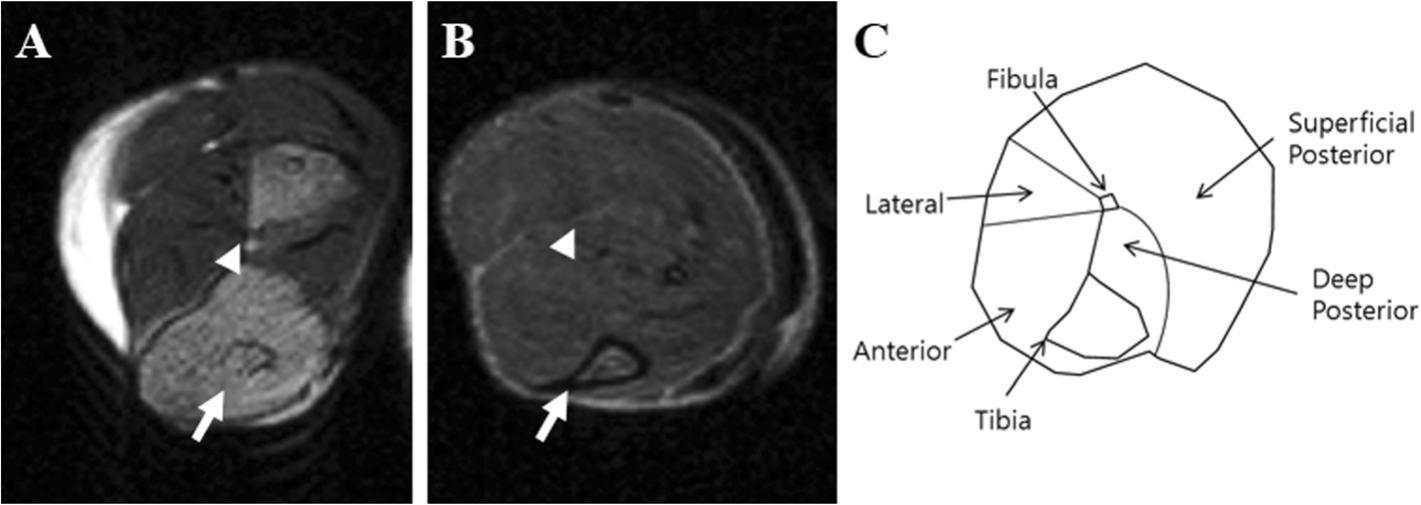
Fatty replacement of calf muscles in HSP27 transgenic mice on T2-weighted MRI. **a**. Fatty infiltration of leg muscles was observed in transgenic mice with HSP27 mutations on MRI. **b**. Normal muscular findings of control mice were shown on MRI. **c**. Orientation and anatomy of calf cross sections. Anterior muscle groups (anterior) include tibialis anterior, extensor halluces longus, and extensor digitorum longus. Lateral muscle groups (lateral) include peroneus longus and peroneus brevis. Deep posterior groups (deep posterior) include tibialis posterior and flexor digitorum longus. Superficial posterior groups (superficial posterior) include gastrocnemius and soleus. The tibial and fibular bones are marked as arrow and arrowhead, respectively

T2-weighted imaging and fat-suppressed imaging of leg and thigh muscles were performed on 7 month-old mice. Heterogeneous areas of elevated intensity were visible in all HSP27-S135F transgenic mice (Fig. 2a) in contrast to the more uniform and dark signal for healthy control muscle tissue (Fig. 2b). Transgenic mice showed marked fatty infiltration in the anterior and posterior compartments at the calf level.

### HSP27-S135F transgenic mice exhibit reduced CMAP

**Fig. 3:**
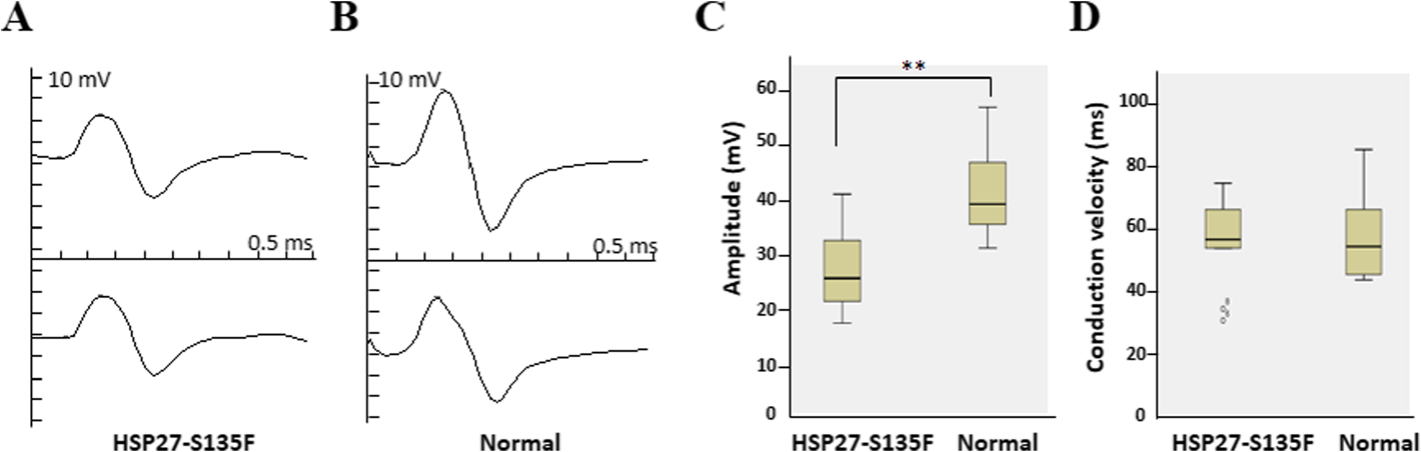
Reduced CMAP was observed from NCS. Representative sciatic CMAP responses from HSP27-S135F (**a**) and normal mice (**b**). Sweep speed, 0.5 ms per division; Amplitude, 10 mV per division. Mean sciatic CMAP amplitudes (**c**) and conduction velocity (**d**) for each group revealed that HSP27-S135F exhibit abnormality in only CMAP. Five mice of each group were used and data from both right and left were pooled (*n* = 10). **, *p* < 0.01

To determine the functional defect in hind limbs, nerve conduction was examined in both sides of sciatic nerve of five F1 mice of the #11 founder at 7 months of age. Five age-matched normal mice were used as control. As shown in Fig. 3a-c and Table 1, transgenic mice showed significantly reduced CMAP than control mice. However, there was no difference in MNCV between normal and HSP27-S135F mice (Table 1 and Fig. 3). These data suggest that HSP27-S135F transgenic mice have abnormal motor function and the phenotype is consistent with CMT2 or dHMN, rather than CMT1, which is caused by Schwann cell malfunction and shows reduced MNCV.

**Table 1:** Summary of nerve conduction study

	Control	HSP27-S135F	*p*-value
n^a^	10	10	-
CMAP	41.27. ± 7.51	27.48 ± 8.21	0.004
MNCV	57.92 ± 14.28	56.50 ± 13.95	0.912

^a^ *n* = sum of left and right sciatic nerve; data expressed as mean ± standard deviation

### Electron microscopy demonstration of reduced motor neuron

**Fig. 4:**
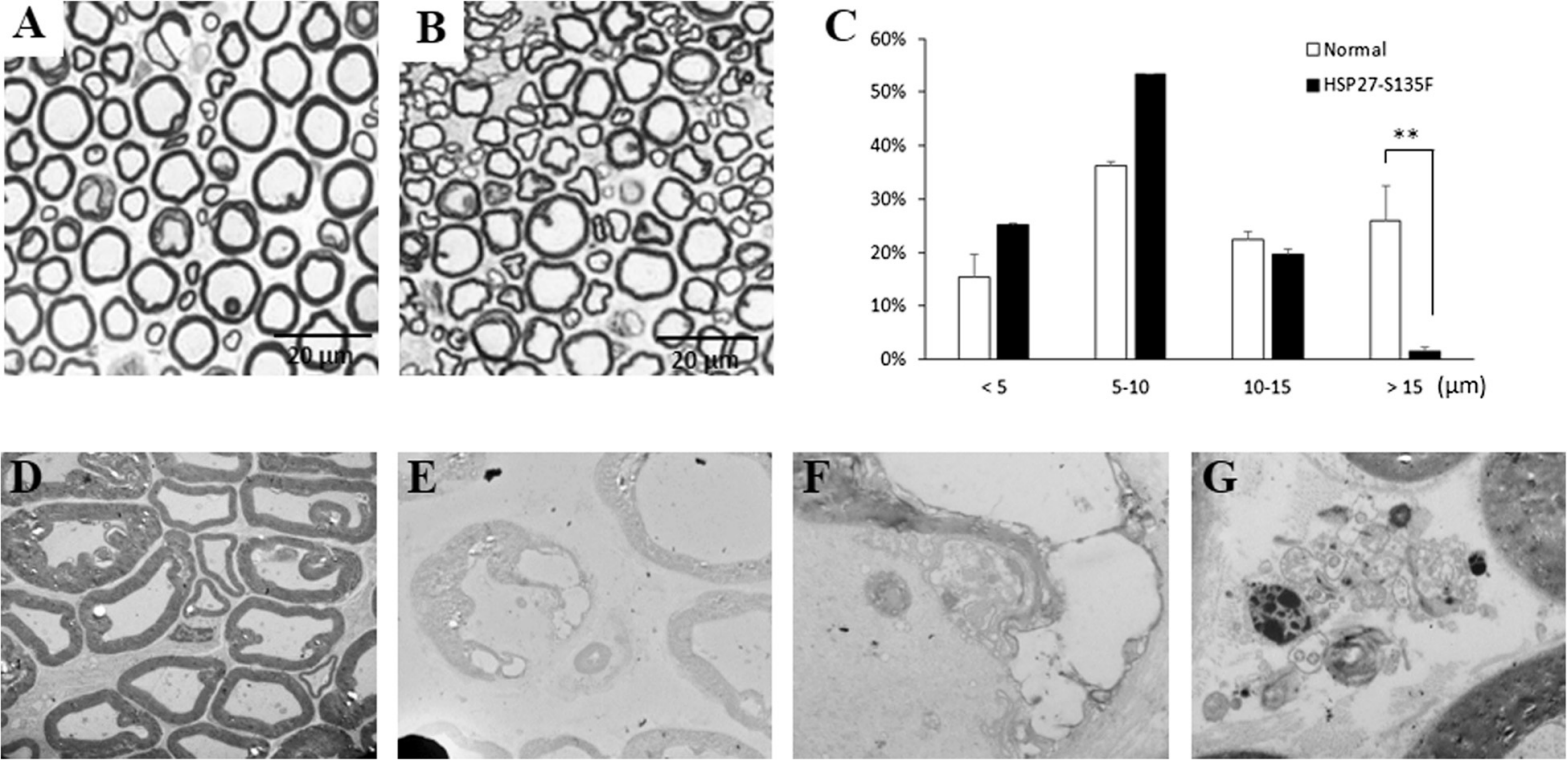
Aberration in myelination was observed in HSP27-S135F mice. Toluidine blue staining showed normal myelination in normal mice (**a**) and demyelination in HSP27-S135F mice (**b**). Scale bar = 20 μM. **c**. Distribution of diameter of myelinated axon demonstrated that the proportion of large (>15 μm) myelinated fibers are reduced in HSP27-S135F mice. **, *p* < 0.01. Electron microscopy demonstrated normal pattern in normal mice (**d**) and abnormal myelination in transgenic mice (**e-g**). Magnifications: (**d**) and (**e**), x 1000; (**f**) and (**g**), x 4000

To analyze the ultra-structure of the sciatic nerve of HSP27-S135F mice, sciatic nerves of the founder and their progeny mice were collected. Semi-thin sections of sciatic nerve displayed reduced number of large myelinated fibers in HSP27-S135F mice compared to control mice (Fig. 4a and 4b). Direct counts also revealed that the ratio of large myelinated fibers (>15 μm) was reduced in transgenic mice compared to control mice (Fig. 4c). Since large myelinated fibers are motor neurons, this result is consistent with previously demonstrated impaired motor function and muscle integrity. Electron microscopic analysis revealed mixed forms of demyelinated or dysmyelinated fibers compared to normal fibers (Fig. 4d-g).

### Decreased level of acetyl-tubulin in sciatic nerve

**Fig. 5:**
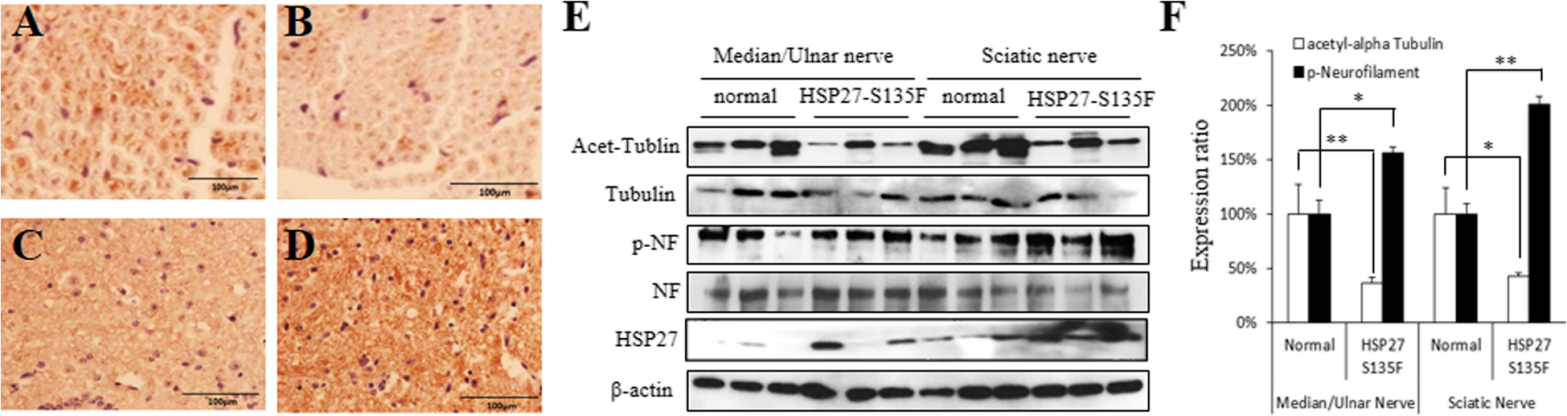
Reduced acetylated tubulin and increased phosphorylated neurofilament. **a**-**d**. Immunohistochemical analyses revealed the reduced level of acetylated tubulin in HSP27-S135F mice (**b**) compared to normal mice (**a**) and elevated level of phosphorylated neurofilament in HSP27-S135F mice (**d**) compared to normal mice (**c**). Scale bar = 100 μM. **e**. Western blotting from 3 mice from each group demonstrated the changes of the proteins level. **f**. The Western blotting image were quantitated. Data are mean ± standard deviation. *, *p* < 0.05; and **, *p* < 0.01

Since phosphorylated neurofilament and acetylated tubulin have been reported as molecular pathological markers for HSP27 mutation mediated axonopathy, we determined their levels in the sciatic nerve. As expected, tubulin acetylation was reduced and phosphorylated neurofilament was elevated in sciatic nerve of HSP27-S135F mice compared to control mice (Fig. 5a-d). Western blotting from each of the three mice (Fig. 5e) and quantitation from the data (Fig. 5f) were also consistent with the immunohistochemical data.

## Discussion

Transgenic mice overexpressing HSP27-S135F were generated and phenotypic characteristics were assessed. The mice exhibited axonal neuropathy as expected. Electron microscopy and toluidine blue staining revealed deteriorated integrity of Schwann cells and axons in transgenic mice. However, the major pathogenesis occurred in the axon because only CMAP rather than MNCV was affected. MNCV is lowered by the aberrant myelination of Schwann cell, which is a typical pattern of CMT1, whereas CMAP is affected by axonal neuropathy, such as CMT2. These axonal defects increase fatty infiltration in the anterior and posterior compartments of the calf, which eventually led to reduced locomotion function. Since HSP27-S135F mutation cause both CMT2F and dHMN, we tried to examine whether the mice exhibit sensory neuropathy. From hot plate test, we could observe that HSP27-S135F mice show normal sensory function on temperature (Additional file 3: Figure S3). Thus phenotypic characteristics of our new mice might be close to dHMN.

Compared to previously reported mouse models, the newly developed model exhibited early onset and more severe phenotype. In a prior study, rotarod performance deteriorated from 6 months of age [18]; however, the newly developed mice showed poor performance from 5 months. Moreover, the performance in two founders was only 55 % and 23 % of control mice, respectively. These observations imply that the phenotype of the mice with the same mutation can be diverse according to the genetic background and driving promoter. In addition, phenotypic diversity could be derived by the position or copy number of the transgene. The phenotypic variation might also be dependent on the expression level of the transgene. We tried to correlate the expression level and the phenotypic severity (Additional file 4: Figure S4), however, further study is needed to provide clear conclusion.

MRI was used to assess muscle integrity in the CMT mouse model. Since the pathological findings from MRI are consistent with previous reports [4], MRI can be adopted as a new assessment tool for CMT research. In CMT, huge gaps still remain between preclinical study and clinical outcomes. Although the administration of ascorbic acid can dramatically reduce the mouse phenotype [22], the effects remain unclear even after phase III clinical trial [23].

The HSP27-S135F missense mutation is located in the HSP20-α-crystallin domain. This domain is related to mitochondrial movement, which is important to maintain the integrity of cellular cytoskeleton transport [18, 24]. HSP27-S135F mutation induces hyper-phosphorylation of neurofilament light protein, resulting in axonal transport defect by aggregation of phosphorylated neurofilament proteins, which is mediated by Cyclin-dependent kinase (Cdk5) [25]. Since elevated level of phospho-NEFL was reduced by inhibitor or by the Cdk5-specific shRNA and the reduced mitochondrial movement, which is mediated by reduced acetylated-α-tubulin, was recovered by the utilization of HDAC inhibitor [18], targeting Cdk5 or HDAC could be the therapeutic strategies for the treatment of axonal neuropathy caused by the mutation in HSP27.

## Conclusion

In conclusion, the phenotype of the newly developed mouse model is consistent with CMT2 or dHMN patients. The model is a potentially useful tool for future assessment of therapeutic candidates for CMT2 or dHMN.

## Additional files

Additional file 1: Figure S1. Generation and expression of HSP27-S135F. (A) Chromatograms of the sequences of wild type and S135F mutants of HSP27. Arrows indicate the mutation site. (B) Western blotting for the determination of the expression wild type and S135F mutant HSP27 in HEK293 cells or NSC34 cells. Additional file 2: Figure S2. Grip strength test for offspring of #11 and #26. Grip strength was performed using all four limbs of the mice. *, *p* < 0.05. Additional file 3: Figure S3. Hot plate test for sensory nerve function. Mice were place on a preheated (52 °C) acrylic box. Latency of paw withdrawal, shaking, or licking was calculated and compared (*n* = 7 per each group). Trembler J mice is well-known CMT1 mouse model, which naturally carries PMP22-L16P mutation in one allele. **, *p* < 0.01. Additional file 4: Figure S4. Expression of HSP27 in the siblings. (A) CMAP were performed to divide the sibling into moderate and severe phenotype. #201, #243, and #293 mice showed relatively mild phenotype, and #217, #240, and #280 mice showed relatively severe phenotype. (B) Western blotting for determination of HSP27 expression level in the sciatic nerve of the mice. (C) Determination of HSP27 expression in the various organs including sciatic nerve, spinal cord, spleen, and liver from normal and HSP27-S135F transgenic mice. Additional file 5:
**Supplementary Methods.**

## Abbreviations

HSP27: Heat shock 27 kDa protein 1
CMT2F: Charcot-Marie-Tooth disease type2F
dHMN: Distal hereditary motor neuropathy
MRI: Magnetic resonance image
CMAP: Compound motor action potential
MNCV: Motor nerve conduction velocity
DRG: Dorsal root ganglion
