# Supplementary figures and images for: Overexpression of mutant HSP27 causes axonal neuropathy in mice
Source: J Biomed Sci. 2015 Jun 19;22(1):43. doi: 10.1186/s12929-015-0154-y (PMC4490621; doi:10.1186/s12929-015-0154-y)

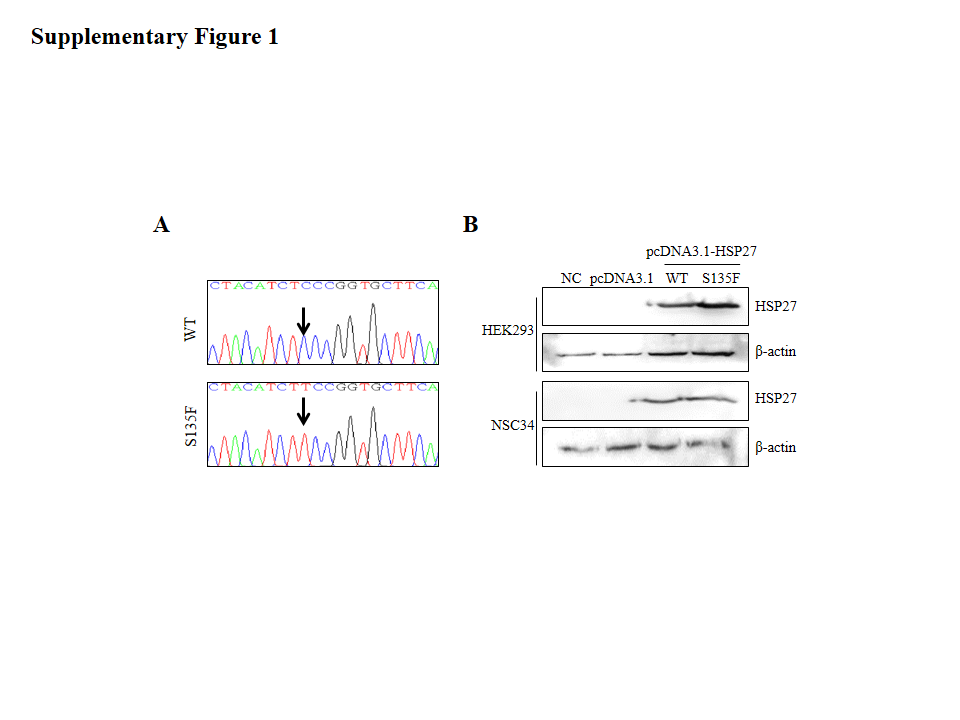

Supplement: Additional file 1: Figure S1. — Generation and expression of HSP27-S135F. (A) Chromatograms of the sequences of wild type and S135F mutants of HSP27. Arrows indicate the mutation site. (B) Western blotting for the determination of the expression wild type and S135F mutant HSP27 in HEK293 cells or NSC34 cells. [file 12929_2015_154_MOESM1_ESM.tif]

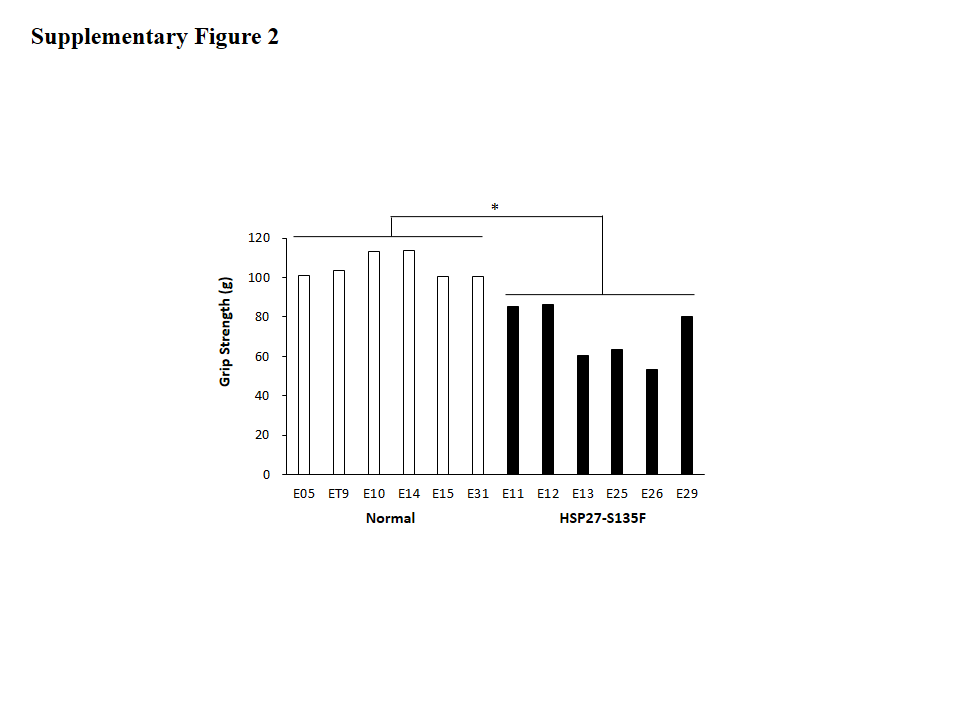

Supplement: Additional file 2: Figure S2. — Grip strength test for offspring of #11 and #26. Grip strength was performed using all four limbs of the mice. *, p < 0.05. [file 12929_2015_154_MOESM2_ESM.tif]

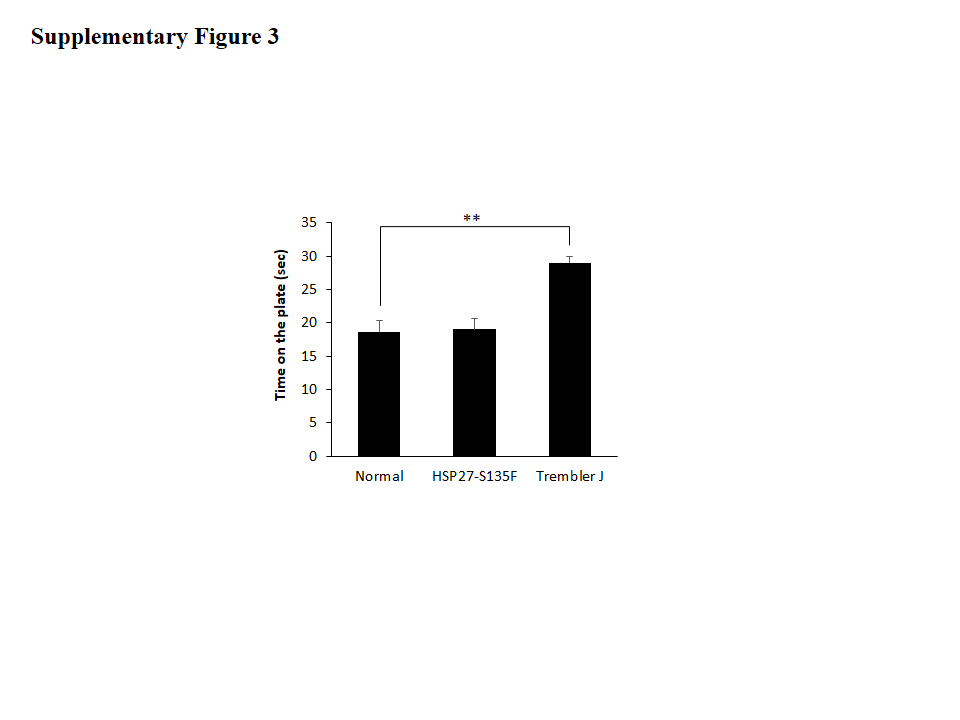

Supplement: Additional file 3: Figure S3. — Hot plate test for sensory nerve function. Mice were place on a preheated (52 °C) acrylic box. Latency of paw withdrawal, shaking, or licking was calculated and compared (n = 7 per each group). Trembler J mice is well-known CMT1 mouse model, which naturally carries PMP22-L16P mutation in one allele. **, p < 0.01. [file 12929_2015_154_MOESM3_ESM.tif]

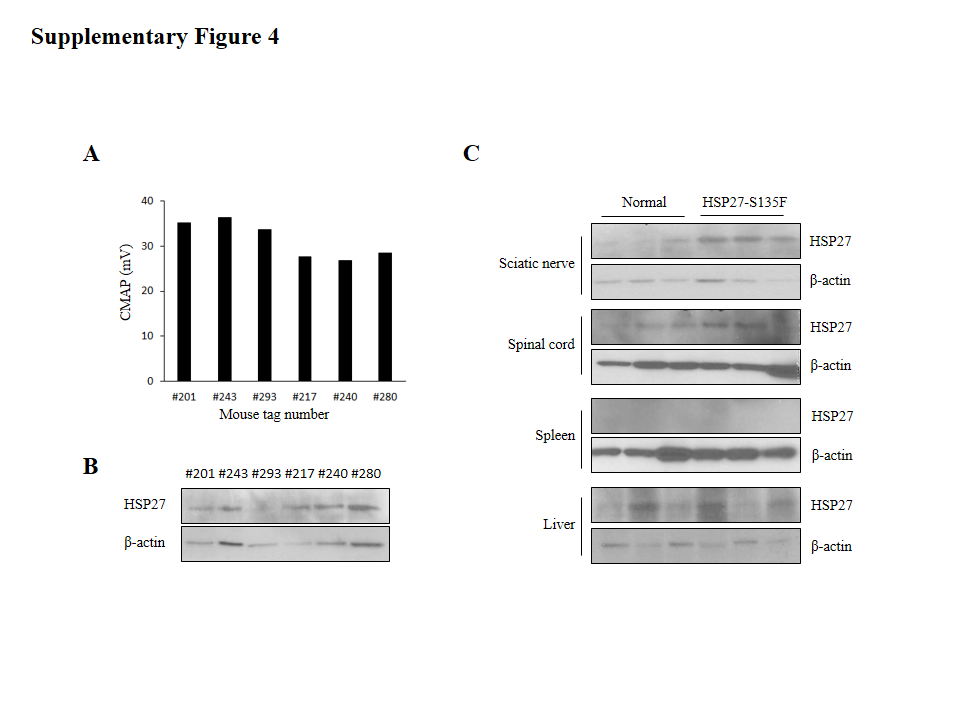

Supplement: Additional file 4: Figure S4. — Expression of HSP27 in the siblings. (A) CMAP were performed to divide the sibling into moderate and severe phenotype. #201, #243, and #293 mice showed relatively mild phenotype, and #217, #240, and #280 mice showed relatively severe phenotype. (B) Western blotting for determination of HSP27 expression level in the sciatic nerve of the mice. (C) Determination of HSP27 expression in the various organs including sciatic nerve, spinal cord, spleen, and liver from normal and HSP27-S135F transgenic mice. [file 12929_2015_154_MOESM4_ESM.tif]
