# Supplementary material for: Overexpression of mutant HSP27 causes axonal neuropathy in mice
Source: J Biomed Sci. 2015 Jun 19;22(1):43. doi: 10.1186/s12929-015-0154-y (PMC4490621; doi:10.1186/s12929-015-0154-y)
Supplement: Additional file 5: — Supplementary Methods. [file 12929_2015_154_MOESM5_ESM.docx]

**Additional file 5**

**MATERIALS AND METHODS**

**Western blotting** To confirm the expression of pcDNA3.1 (+)-*HSP27* wild type and S135F construct, NSC-34 cells (3×10^5^ cells/well) and HEK 293 cells (2×10^5^ cells/well) were seeded on 6 well plate for 24 hrs. Cloned genes were transfected into HEK 293 and NSC-34 cells for 24 hrs. Expression of the HSP27 protein was detected with anti-HSP27 Antibody and donkey anti-goat secondary antibody (Santa Cruz Biotechnology, Santa Cruz, CA, USA).

**Genotyping of HSP27 transgenic mice** Genomic DNA of transgenic mice was extracted at postnatal day 21. Genotyping of *HSP27* transgenic mice were carried out using Extract-N-Amp^TM^ Tissue PCR Kit (Sigma-Aldrich, St. Louis, MO) according to vender’s protocol with the following primer set: CMV-HSP27-F, 5’-GACGTCAATGGGAGTTTGTTTT-3’; and CMV-HSP27-R, 5’-GAGATGTAGCCATGCTCGTCCT-3’.
